# Optimized Multi-Position Calibration Method with Nonlinear Scale Factor for Inertial Measurement Units

**DOI:** 10.3390/s19163568

**Published:** 2019-08-15

**Authors:** Zihui Wang, Xianghong Cheng, Jinbo Fu

**Affiliations:** 1 School of Instrument Science and Engineering, Southeast University, Nanjing 210096, China; 2 Key Laboratory of Micro-Inertial Instrument and Advanced Navigation Technology, Ministry of Education, Southeast University, Nanjing 210096, China

**Keywords:** inertial measurement units, nonlinear scale factor, multi-position method, optimization method

## Abstract

Navigation grade inertial measurement units (IMUs) should be calibrated after Inertial Navigation Systems (INSs) are assembled and be re-calibrated after certain periods of time. The multi-position calibration methods with advantage of not requiring high-precision equipment are widely discussed. However, the existing multi-position calibration methods for IMU are based on the model of linear scale factors. To improve the precision of INS, the nonlinear scale factors should be calibrated accurately. This paper proposes an optimized multi-position calibration method with nonlinear scale factor for IMU, and the optimal calibration motion of IMU has been designed based on the analysis of sensitivity of the cost function to the calibration parameters. Besides, in order to improve the accuracy and robustness of the optimization, an estimation method on initial values is presented to solve the problem of setting initial values for iterative methods. Simulations and experiments show that the proposed method outperforms the calibration method without nonlinear scale factors. The navigation accuracy of INS can be improved by up to 17% in lab conditions and 12% in the moving vehicle experiment, respectively.

## 1. Introduction

Inertial Navigation System (INS) is an entirely self-contained system that solves positions of a point by integrating its accelerations [1]. It can provide high-rate attitude, velocity and position information, hence it is widely used as the navigation means of autonomous underwater vehicles, missiles, robots, aircrafts and other autonomous vehicles. Inertial Measurement Unit (IMU), composed of accelerometer and gyroscope, is the essential device of INS that plays a critical role in the precision of INS [2]. IMU calibration is a process of estimating the coefficients that convert the raw outputs of IMU to accelerations and rotation rates, which can be used to reflect the motion status of the vehicle. The applications of IMU calibration can be divided into two types: the factory calibration after the navigation system is assembled and the re-calibration process after certain intervals of time. 

Traditionally, the navigation-grade IMU calibration is performed by comparing the IMU outputs with a known reference generated from the high-precision equipment [3]. Apparently, the accuracy of the traditional methods strongly relies on the accuracy of the calibration equipment [4]. Due to the frequent unavailability of costly high-precision calibration equipment, the multi-position calibration method has been widely discussed in recent years [5,6,7,8,9,10,11,12,13,14], which is based on the principle that the norms of outputs of the accelerometer and the gyroscope clusters are equal to the magnitudes of inputs of specific force and rotational velocity respectively. The multi-position calibration method can date back to 1995 when Ferraris F et al conducted their research in which the accelerometers and gyroscopes are calibrated by the local gravity and the geometrical quantities respectively without any rotation table or other velocity standards [5]. The IMU is placed on six static positions to determine the biases and scale factors, but unable to obtain the misalignment errors. Shin and Sheimy extended the methods by estimating the misalignment errors with gravity and earth rotation rate in 2002 [6]. However, the main drawback of this method is that the gyroscope scale factors and misalignment errors cannot be estimated reliably because of the observable problem that the magnitude of the earth rotation rate is very small. The method was modified in 2006 to calibrate the misalignment errors of gyroscopes with a single-axis turntable to provide a strong rotation rate signal by Newton’s method [7]. David Jurman et al in 2007 proposed a method which employs constrained Newton optimization procedure for the estimation of 9 parameters including the bias, scale factor errors and misalignment errors [8]. However, this method suffers from the disadvantage of large computation and demand on precise initial conditions. 

Zhang et al in 2009 transformed the optimal problem to a set of linear equations and proposed a new approach to calibrate the inter-triad misalignment [9]. Although this method do not need any initial values like other iterative method the number of position and rotation clusters for IMU should be more than the number of equations in order to solve the set of equations. Cheuk et al in 2012 proposed a hand motion-based method to calibrate the consumer-grade IMU and utilized the accelerometer and magnetometer as the reference to calibrate the gyroscope in six minutes [10]. Yang et al in 2012 proposed an improved iterative estimation method to derive the scale factors, misalignments, biases and squared coefficients without any orientation information [11]. Cai et al in 2013 presented a calibration method for accelerometer with nonlinear scale factor using the particle swarm optimization to solve the nonlinear equation [12]. Although the results are very promising, the problem of initial values setting still exists. Särkkä et al in 2017 proposed an enhanced multi-position calibration method based on Gauss-Newton method for consumer-grade accelerometers, gyroscopes and magnetometers, and for accelerometers and magnetometers, the direction of reference signals, such as the gravity and the magnetic field of the Earth, are estimated with calibration parameters [13]. Wang et al in 2017 presented a 16-position calibration method for gyroscope’s drift coefficients on centrifuge [14]. 

The calibration process is an optimized estimation for parameters. There are many methods for solving the following unconstrained optimization problem

(1) $$\underset{x\in R^{n}}{\min}f\left(x\right)$$

where *f*(***x***) is a smooth cost function. Among them the line search methods [15,16,17] and trust region methods [18,19,20] are the most popular ones. Levenberg-Marquardt (LM) optimization with trust region method makes a good trade-off between the steepest decent method and the Gauss-Newton method which is widely used in nonlinear optimization [21,22]. However, the initial values should be approximately determined before estimation [23].

In a word, although the multi-position calibration method has been widely researched, some problems are still not settled. Firstly, the nonlinear scale factor of gyroscope and accelerometer should be calibrated accurately to improve the precision of INS. IMU has different scale factor for different specific force and angular velocity, due to the change of temperature and magnetic field. Besides, the scale factor often changes as a function of the products of specific force and angular velocity components [12]. The optimization methods used in the literature can be divided into 3 types: (1) Transform the set of nonlinear equations into linear equations; (2) Use the iterative methods and (3) Use the particle swarm optimization. However, the number of position and rotation for IMU should be increased to solve the linear equations in method (1). Rough initial values are needed to obtain global optimal values in method (2). The training process is cumbersome in method (3). Secondly, a simple optimization method without those disadvantages in method (1–3) should be investigated. 

The main purpose of this paper is to improve the accuracy of IMU by estimating its nonlinear coefficients. The rest of this paper is organized as follows. The IMU models designed in this paper are discussed in Section 2, including the nonlinear scale factors. The effects of nonlinear scale factors of IMU on the performance of INS are discussed in Section 3. The nonlinear optimization methods based on the optimal motions of IMU and the nonlinear optimization method with initial values estimation are discussed in Section 4. The analysis of simulation and experiment results are presented in Section 5. Finally, the conclusions are concluded in Section 6.

## 2. IMU Model

### 2.1. Definition of Frames

The frames used in the paper are listed in Table 1.

**Figure 1:**
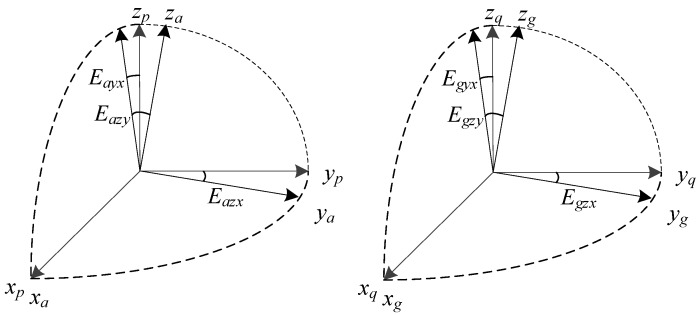
Definition of the *p*-frame and *q*-frame.

A six-degree-of-freedom IMU composed of a triple axis accelerometer and a triple axis gyroscope. The accelerometer senses the acceleration along each input axis in *a*-frame, while gyroscope measures the angular velocity around each input axis in *g*-frame. Due to the assembly imperfections, each axis of IMU deviates by a small angle from its designed mounting direction. Hence, both *a*-frame and *g*-frame are the non-orthogonal frames. The *p*-frame is defined as follows: axis *x_p_* of *p*-frame coincides with the unit vector *x_a_* of *a*-frame, axis *y_p_* is perpendicular to *x_p_* in the plane *x_a_y_a_*, while *z_p_*, *x_p_* and *y_p_* together form the right-hand frame. The *q*-frame is defined as follows: axis *x_q_* of *q*-frame coincides with the unit vector *x_g_* of *g*-frame, axis *y_q_* is perpendicular to *x_q_* in the plane *x_g_y_g_*, while *z_q_*, *x_q_* and *y_q_* together form the right-hand frame. The definition of *p*-frame and *q*-frame are shown in Figure 1, and *E_ayx_*, *E_azx_*, *E_azy_*, *E_ayx_*, *E_azx_*, *E_azy_* are the misalignment errors of IMU. The transformation matrix $C_{p}^{q}$ is defined as the inter-triad misalignment, which represent the transform form *p*-frame to *q*-frame.

### 2.2. Nonlinear Model of Accelerometers

In this paper, the nonlinear model of accelerometers is established inspired by reference [12], and the accelerometer model is expressed as

(2) $$f^{p}=C_{a}^{p}\left(K_{a1}N^{a}+K_{a2}N^{a}{}^{\left(2\right)}+K_{a3}N^{a}{}^{\left(3\right)}+\nabla^{a}\right)+v^{p}\overset{def}{=}{\tilde{f}}^{p}+v^{p}\overset{def}{=}C_{a}^{p}{\tilde{f}}^{a}+v^{p}$$

where *f^p^* is the true value of the specific force in *p*-frame, ${\tilde{f}}^{p}$ and ${\tilde{f}}^{a}$ are the raw output of accelerometers in *p*-frame and *a*-frame, respectively; ***K****_a_*_1_ = diag(*K_ax_*_1_, *K_ay_*_1_, *K_az_*_1_), ***K****_a_*_2_ = diag(*K_ax_*_2_, *K_ay_*_2_, *K_az_*_2_) and ***K****_a_*_3_ = diag(*K_ax_*_3_, *K_ay_*_3_, *K_az_*_3_) are the linear, the second-order and the third-order scale factor of accelerometers, respectively; **▽***^a^* and ***v**^p^* are the bias and the noise of accelerometers, respectively; ***N**^a^* is the output of accelerometers in pulses per second, ***N**^a^*^(2)^ denotes the vector with the square of each element in ***N****^a^* and ***N****^a^*^(3)^ denotes the vector with the cube of each element in ***N****^a^*:

(3) $$N^{a}{}^{\left(2\right)}={\left[\begin{array}{ccc} {\left(N_{x}^{a}\right)}^{2} & {\left(N_{y}^{a}\right)}^{2} & {\left(N_{z}^{a}\right)}^{2} \end{array}\right]}^{T}$$

(4) $$N^{a}{}^{\left(3\right)}={\left[\begin{array}{ccc} {\left(N_{x}^{a}\right)}^{3} & {\left(N_{y}^{a}\right)}^{3} & {\left(N_{z}^{a}\right)}^{3} \end{array}\right]}^{T}$$

$C_{a}^{p}$ is the transformation matrix from *a*-frame to *p*-frame, and can be written as

(5) $$C_{a}^{p}=\left(\begin{array}{ccc} 1 & 0 & 0\\ E_{ayx} & 1 & 0\\ E_{azx} & E_{azy} & 1 \end{array}\right)$$

where *E_ayx_*, *E_azx_*, *E_azy_* are the misalignment errors of accelerometers.

### 2.3. Nonlinear Model of Gyroscopes

Similarly, the gyroscope model can be expressed as

(6) $$\omega_{iq}^{q}=C_{g}^{q}\left(K_{g1}N^{g}+K_{g2}N^{g}{}^{\left(2\right)}+\varepsilon^{g}+v^{g}\right)$$

where $\omega_{iq}^{q}$ is the true value of gyroscope output in *q*-frame, ***K****_g_*_1_ = diag(*K_gx_*_1_, *K_gy_*_1_, *K_gz_*_1_) and ***K****_g_*_2_ = diag(*K_gx_*_2_, *K_gy_*_2_, *K_gz_*_2_) are the linear and the second-order scale factor of gyroscopes, respectively; ***ε****^g^* and ***v****^g^* are the bias and the noise of gyroscopes, respectively; ***N****^g^* is the raw output of gyroscopes in pulses per second, ***N****^g^*^(2)^ denotes the vector with the square of each element in ***N****^g^*:

(7) $$N^{g}{}^{\left(2\right)}={\left[\begin{array}{ccc} {\left(N_{x}^{g}\right)}^{2} & {\left(N_{y}^{g}\right)}^{2} & {\left(N_{z}^{g}\right)}^{2} \end{array}\right]}^{T}$$

$C_{g}^{q}$ is the transformation matrix from *g*-frame to *q*-frame, and can be written as

(8) $$C_{g}^{q}=\left(\begin{array}{ccc} 1 & 0 & 0\\ E_{gyx} & 1 & 0\\ E_{gzx} & E_{gzy} & 1 \end{array}\right)$$

where *E_gyx_*, *E_gzx_*, *E_gzy_* are the misalignment errors of gyroscopes.

The task of IMU calibration is to estimate the linear scale factors, nonlinear scale factors, biases and the misalignments of IMU. According to (2) and (6), the raw output of IMU can be calibrated in the orthogonal *p*-frame and *q*-frame from non-orthogonal *a*-frame and *g*-frame, respectively. In order to ensure the accuracy of alignment and navigation, it is necessary to calibrate the accelerometer and gyroscope in the same frame. Hence, the calibration method of the inter-triad misalignment in Reference [9] is utilized in this paper.

## 3. The Effects of Nonlinear Scale Factors

For high-precision navigation grade IMU with the precision of 0.01 mg and 0.01 °/h, the nonlinear scale factors should not be ignored. This section discuses about the effects of Nonlinear Scale Factors of IMU on the precision of INS. The well-known differential equations of navigation error for INS are described as [24]:

(9) $$\delta {\dot{V}}^{n}=f^{n}\times \phi^{n}+\delta V^{n}\times \left(2\omega_{ie}^{n}+\omega_{en}^{n}\right)+V^{n}\times \left(2\delta \omega_{ie}^{n}+\delta \omega_{en}^{n}\right)+\delta f^{n}$$

(10) $${\dot{\phi }}^{n}=\phi^{n}\times \left(\omega_{ie}^{n}+\omega_{en}^{n}\right)+\delta \omega_{ie}^{n}+\delta \omega_{en}^{n}-\delta \omega_{ib}^{n}$$

where ***V****^n^* = [*V_E_ V_N_ V_U_*]^T^ denotes the velocity of vehicle in *n*-frame, ***f****^n^* = [*f_E_ f_N_ f_U_*]^T^ denotes the accelerometers output in *n*-frame and ***ϕ*** = [*ϕ_E_ ϕ_N_ ϕ_U_*]^T^ denotes the misalignment angle between *n*-frame and *n*′-frame. *δ*[·] denotes the error of the vector [·]. The rotation rate of the earth in *n*-frame is

(11) $$\omega_{ie}^{n}={\left[0\;\omega_{ie}\cos L\;\omega_{ie}\sin L\right]}^{T}$$

where *L* denotes the latitude of the vehicle, and the rotation rate of *n*-frame related to *e*-frame is

(12) $$\omega_{en}^{n}={\left[-\frac{V_{N}}{R_{M}}\;\frac{V_{E}}{R_{N}}\;\frac{V_{E}}{R_{N}}\tan L\right]}^{T}$$

where *R_M_* and *R_N_* denote the meridian radius of the earth. In order to analysis the effects of nonlinear scale factors of IMU only, substitute Equations (2), (6) into Equations (9), (10) and we have,

(13) $$\delta {\dot{V}}^{n}=f^{n}\times \phi^{n}+\delta V^{n}\times \left(2\omega_{ie}^{n}+\omega_{en}^{n}\right)+V^{n}\times \left(2\delta \omega_{ie}^{n}+\delta \omega_{en}^{n}\right)+C_{b}^{n}\left(K_{a2}N^{a}{}^{\left(2\right)}+K_{a3}N^{a}{}^{\left(3\right)}\right)$$

(14) $${\dot{\phi }}^{n}=\phi^{n}\times \left(\omega_{ie}^{n}+\omega_{en}^{n}\right)+\delta \omega_{ie}^{n}+\delta \omega_{en}^{n}-C_{b}^{n}K_{g2}N^{g}{}^{\left(2\right)}$$

Hence, if the nonlinear scale factors are not calibrated, the velocity and attitude errors of INS are increased as shown in Equations (13) and (14). There exists a constant error in the differential equations caused by the nonlinear scale factors of IMU. Besides, when the vehicle is in the quasi-static state, the navigation error equations can be simplified as

(15) $$\delta {\dot{V}}^{n}=f^{n}\times \phi^{n}+C_{b}^{n}\left(K_{a2}N^{a}{}^{\left(2\right)}+K_{a3}N^{a}{}^{\left(3\right)}\right)$$

(16) $${\dot{\phi }}^{n}=\phi^{n}\times \omega_{ie}^{n}-C_{b}^{n}K_{g2}N^{g}{}^{\left(2\right)}$$

It can be seen that as time grows, the error caused by the nonlinear scale factors will be integrated into the velocity and attitude errors of INS. In summary, the nonlinear scale factors of IMU determines the growth speed of navigation errors. Hence, in order to improve the precision of INS, the nonlinear scale factors should be calibrated accurately.

## 4. Nonlinear Optimization Method

### 4.1. Nonlinear Optimization of Accelerometer

Theoretically, the norm of accelerometer output in *p*-frame is equal to the specific force in *n*-frame, that is:

(17) $${\left\|f^{p}\right\|}^{2}={\left\|G^{n}\right\|}^{2}$$

where ***G*** is the gravity vector. According to (2) and (6), the nonlinear cost function of accelerometer can be expressed as:

(18) $$\underset{K_{a1},K_{a2},K_{a3},C_{a}^{p},\nabla^{a}}{\min}\frac{1}{2}\sum_{i=1}^{m}{\left\|{\left\|C_{a}^{p}\left(K_{a1}N_{i}^{a}+K_{a2}N_{i}^{a\left(2\right)}+K_{a3}N_{i}^{a\left(3\right)}+\nabla^{a}\right)\right\|}^{2}-{\left\|G^{n}\right\|}^{2}\right\|}^{2}$$

where $N_{i}^{a}$ is the output of accelerometer in the *i*th position. 

In order to find the optimized position cluster and identify all parameters of accelerometer, the sensitivity of measurement to the parameters should be maximized. Define

(19) $$\begin{array}{c} F={\left\|C_{a}^{p}\left(K_{a1}N_{i}^{a}+K_{a2}N_{i}^{a\left(2\right)}+K_{a3}N_{i}^{a\left(3\right)}+\nabla^{a}\right)\right\|}^{2}-{\left\|G^{n}\right\|}^{2}={\left\|{\tilde{f}}^{p}\right\|}^{2}-{\left\|G^{n}\right\|}^{2}\\ \;={\left(K_{a1x}N_{x}+K_{a2x}N_{x}^{2}+K_{a3x}N_{x}^{3}+\nabla_{x}^{a}\right)}^{2}+(E_{ayx}K_{a1x}N_{x}+K_{a1y}N_{y}+E_{ayx}K_{a2x}N_{x}^{2}+K_{a2y}N_{y}^{2}\\ \;{\;+E_{ayx}K_{a3x}N_{x}^{3}+K_{a3y}N_{y}^{3}+E_{ayx}\nabla_{x}^{a}+\nabla_{y}^{a})}^{2}+(E_{azx}K_{a1x}N_{x}+E_{azy}K_{a1y}N_{y}+K_{a1z}N_{z}\\ \;+E_{azx}K_{a2x}N_{x}^{2}+E_{azy}K_{a2y}N_{y}^{2}+K_{a2z}N_{z}^{2}+E_{azx}K_{a3x}N_{x}^{3}+E_{azy}K_{a3y}N_{y}^{3}+K_{a3z}N_{z}^{3}\\ \;{+\;E_{azx}\nabla_{x}^{a}+E_{azy}\nabla_{y}^{a}+\nabla_{z}^{a})}^{2}-g^{2} \end{array}$$

Take the partial derivatives of (19) with respect to the linear scale factor *Ka*_1_*x*, and ignore the little quantity items with misalignment errors:

(20) $$\frac{\partial F}{\partial K_{a1x}}=2{\tilde{f}}_{x}^{p}N_{x}+2{\tilde{f}}_{y}^{p}E_{ayx}N_{x}+2{\tilde{f}}_{z}^{p}E_{azx}N_{x}\approx 2{\tilde{f}}_{x}^{p}N_{x}$$

Similarly, take the partial derivatives of (19) with respect to other parameters:

(21) $$\frac{\partial F}{\partial K_{a1y}}=2{\tilde{f}}_{y}^{p}N_{y},\;\frac{\partial F}{\partial K_{a1z}}=2{\tilde{f}}_{z}^{p}N_{z}$$

(22) $$\frac{\partial F}{\partial K_{a2x}}=2{\tilde{f}}_{x}^{p}N_{x}^{2},\;\frac{\partial F}{\partial K_{a2y}}=2{\tilde{f}}_{y}^{p}N_{y}^{2},\;\frac{\partial F}{\partial K_{a2z}}=2{\tilde{f}}_{z}^{p}N_{z}^{2}$$

(23) $$\frac{\partial F}{\partial K_{a3x}}=2{\tilde{f}}_{x}^{p}N_{x}^{3},\;\frac{\partial F}{\partial K_{a3y}}=2{\tilde{f}}_{y}^{p}N_{y}^{3},\;\frac{\partial F}{\partial K_{a3z}}=2{\tilde{f}}_{z}^{p}N_{z}^{3}$$

(24) $$\frac{\partial F}{\partial \nabla_{x}^{a}}=2{\tilde{f}}_{x}^{p},\;\frac{\partial F}{\partial \nabla_{y}^{a}}=2{\tilde{f}}_{y}^{p},\;\frac{\partial F}{\partial \nabla_{z}^{a}}=2{\tilde{f}}_{z}^{p}$$

(25) $$\frac{\partial F}{\partial E_{ayx}}=2{\tilde{f}}_{y}^{p}{\tilde{f}}_{x}^{a},\;\frac{\partial F}{\partial E_{azx}}=2{\tilde{f}}_{z}^{p}{\tilde{f}}_{x}^{a},\;\frac{\partial F}{\partial E_{azy}}=2{\tilde{f}}_{z}^{p}{\tilde{f}}_{y}^{a}$$

**Figure 2:**
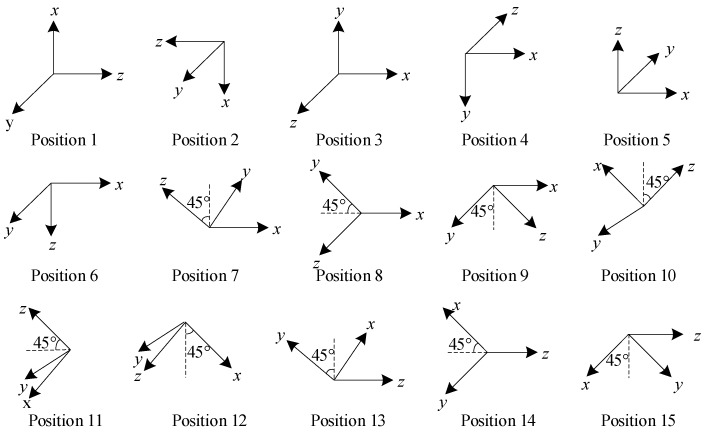
The 15 optimal position clusters.

Based on Equations (20)–(25), the sensitivity of measurement to the parameters can be analyzed. Firstly, the sensitivity to linear scale factor, nonlinear scale factors and bias of the *i*-axis (*i* = *x*, *y*, *z*) accelerometer are maximized, when the sensitive direction of the *i*-axis (*i* = *x*, *y*, *z*)) accelerometer is parallel to the gravity vector. Secondly, the sensitivity to misalignment errors *E_aij_* (*i* = *y*, *j* = *x* or *i* = *z*, *j* = *x*, *y*) is maximized, when the gravity vector is in the plane formed by the *i*-axis and the *j*-axis accelerometer with an angle of 45° or 135° between the axis and the gravity vector. Hence, considering the sensitivity of measurement to parameters, the optimal position clusters to estimate the 15 parameters in (2) are shown in Figure 2. 

### 4.2. Nonlinear Optimization of Gyroscope

The output of gyroscope contains the angular rate of SINS and the earth’s rotation rate, that is:

(26) $$\omega_{iq}^{q}=\omega_{ie}^{q}+\omega_{r}^{q}$$

where $\omega_{iq}^{q}$ is the true value of gyroscope output, $\omega_{ie}^{q}$ is the earth’s rotation rate, $\omega_{r}^{q}$ is the true value of the rotation rate that SINS is sensitive to. Different from the optimization of accelerometer, the earth’s rotation rate is too small for gyroscope calibration of accurate scale factors and misalignment [6]. Hence, clockwise and counter-clockwise rotation of SINS is utilized to estimate the scale factors and misalignment of gyroscope firstly.

Take (6) in (26), and integrate the angular rate over the clockwise rotation period *t*_1_, we have:

(27) $$C_{g}^{q}\int_{0}^{t_{1}}\left(K_{g1}N{\left(\tau \right)}^{g+}+K_{g2}N{\left(\tau \right)}^{g}{}^{(2)+}+\varepsilon^{g}+v^{g}\right)d\tau =\int_{0}^{t_{1}}\omega_{ie}^{q}d\tau +\int_{0}^{t_{1}}\omega_{r}^{q}\left(\tau \right)\;d\tau$$

where ***N***(*τ*)*^g^*^+^ is the output of gyroscope during clockwise rotation. Similarly, when SINS rotates in counter-clockwise during the period of *t*_2_:

(28) $$C_{g}^{q}\int_{0}^{t_{2}}\left(K_{g1}N{\left(\tau \right)}^{g-}+K_{g2}N{\left(\tau \right)}^{g}{}^{(2)-}+\varepsilon^{g}+v^{g}\right)d\tau =\int_{0}^{t_{2}}\omega_{ie}^{q}d\tau -\int_{0}^{t_{2}}\omega_{r}^{q}\left(\tau \right)\;d\tau$$

where ***N***(*τ*)*^g^*^−^ is the output of gyroscope during counter-clockwise rotation. Subtract (28) from (27), and make *t* = *t*_1_ = *t*_2_:

(29) $$C_{g}^{q}K_{g1}\int_{0}^{t}\left(N{\left(\tau \right)}^{g+}-N{\left(\tau \right)}^{g-}\right)d\tau +C_{g}^{q}K_{g2}\int_{0}^{t}\left(N{\left(\tau \right)}^{g(2)+}-N{\left(\tau \right)}^{g(2)-}\right)d\tau =2\theta$$

where $\theta =\int_{0}^{t}\omega_{r}^{q}\left(\tau \right)\;d\tau$ is the rotation angle of SINS in a period of *t*, hence the nonlinear cost function of gyroscope can be expressed as:

(30) $$\underset{K_{g1},K_{g2},C_{g}^{q}}{\min}\frac{1}{2}\sum_{i=1}^{n}{\left\|{\left\|C_{g}^{q}K_{g1}N_{sum}^{i}+C_{g}^{q}K_{g2}N_{sum}^{i\left(2\right)}\right\|}^{2}-{\left\|2\theta \right\|}^{2}\right\|}^{2}$$

where $N_{sum}^{i}=\int_{0}^{t}\left(N{\left(\tau \right)}^{g+}-N{\left(\tau \right)}^{g-}\right)d\tau$ is the integration of gyroscope output in the *i*th rotation, and $N_{sum}^{i}=\int_{0}^{t}\left(N{\left(\tau \right)}^{g\left(2\right)+}-N{\left(\tau \right)}^{g\left(2\right)-}\right)d\tau$.

Similar to accelerometer, in order to find the optimized rotation cluster and then identify the scale factors and the misalignment errors of gyroscope, the sensitivity of measurement to the parameters is maximized. Define

(31) $$\begin{array}{c} P={\left\|C_{g}^{q}K_{g1}N_{sum}^{i}+C_{g}^{q}K_{g2}N_{sum}^{i\left(2\right)}\right\|}^{2}-{\left\|2\theta \right\|}^{2}\overset{def}{=}{\left\|\psi^{q}\right\|}^{2}-{\left\|2\theta \right\|}^{2}\\ \;={\left(K_{g1x}N_{sumx}+K_{g2x}N_{sumx}^{\left(2\right)}\right)}^{2}+(E_{gyx}K_{g1x}N_{sumx}+K_{g1y}N_{sumy}+E_{gyx}K_{g2x}N_{sumx}^{\left(2\right)}\\ \;{\;+K_{g2y}N_{sumy}^{\left(2\right)})}^{2}+(E_{gzx}K_{g1x}N_{sumx}+E_{gzy}K_{g1y}N_{sumy}+K_{g1z}N_{sumz}\\ \;{+\;E_{gzx}K_{g2x}N_{sumx}^{\left(2\right)}+E_{gzy}K_{g2y}N_{sumy}^{\left(2\right)}+K_{g2z}N_{sumz}^{\left(2\right)})}^{2}-{\left\|2\theta \right\|}^{2} \end{array}$$

where ***N****_sum_* = [*N_sumx_*, *N_sumy_*, *N_sumz_*]^T^, and define $\psi^{q}=C_{g}^{q}K_{g1}N_{sum}^{}+C_{g}^{q}K_{g2}N_{sum}^{\left(2\right)}=C_{g}^{q}\psi^{g}$. Take partial derivatives of (31) with respect to the scale factors and misalignment errors:

(32) $$\frac{\partial P}{\partial K_{g1x}}=2\psi_{x}^{q}N_{sumx},\;\frac{\partial P}{\partial K_{g1y}}=2\psi_{y}^{q}N_{sumy},\;\frac{\partial P}{\partial K_{g1z}}=2\psi_{z}^{q}N_{sumz}$$

(33) $$\frac{\partial P}{\partial K_{g2x}}=2\psi_{x}^{q}N_{sumx}^{\left(2\right)},\;\frac{\partial P}{\partial K_{g2y}}=2\psi_{y}^{q}N_{sumy}^{\left(2\right)},\;\frac{\partial P}{\partial K_{g2z}}=2\psi_{z}^{q}N_{sumz}^{\left(2\right)}$$

(34) $$\frac{\partial P}{\partial E_{gyx}}=2\psi_{y}^{q}\psi_{x}^{g},\;\frac{\partial P}{\partial E_{gzx}}=2\psi_{z}^{q}\psi_{x}^{g},\;\frac{\partial P}{\partial E_{gzy}}=2\psi_{z}^{q}\psi_{y}^{g}$$

**Figure 3:**
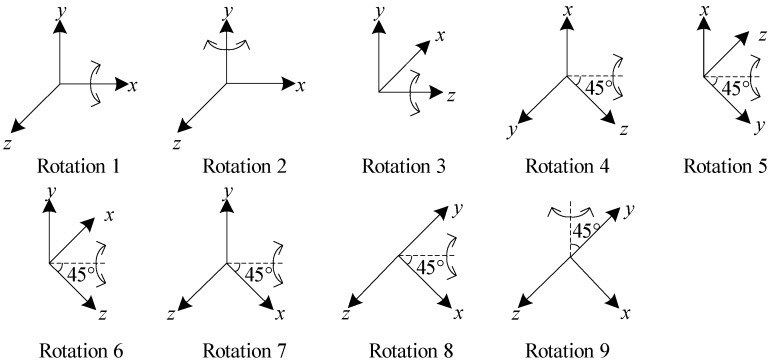
The 9 optimal rotation clusters.

According to equations (32)–(34), the sensitivity to scale factors of the *i*-axis (*i* = *x*, *y*, *z*) gyroscope are maximized, when the sensitive direction of the *i*-axis (*i* = *x*, *y*, *z*) gyroscope is parallel to the rotation vector. Besides, the sensitivity to misalignment errors *E_gij_* (*i* = *y*, *j* = *x* or *i* = *z*, *j* = *x*, *y*) is maximized, when the rotation vector is in the plane formed by the *i*-axis and the *j*-axis gyroscope with an angle of 45° or 135° between the axis and the rotation vector. Hence, the optimization of rotation clusters is to estimate the 9 parameters: the scale factors and misalignment errors in (6) are shown in Figure 3. 

The bias of gyroscope can be estimated using the position clusters redundantly shown in Figure 2, with the cost function:

(35) $$\underset{\varepsilon^{g}}{\min}\frac{1}{2}\sum_{i=1}^{m}{\left\|{\left\|C_{g}^{q}\left(K_{g1}N_{i}^{g}+K_{g2}N_{i}^{g\left(2\right)}+\varepsilon^{g}\right)\right\|}^{2}-{\left\|\omega_{ie}^{n}\right\|}^{2}\right\|}^{2}$$

where $C_{g}^{q}$, $K_{g1}$ and $K_{g2}$ are estimated in (30), $N_{i}^{g}$ is the output of gyroscope in the *i*th position.

### 4.3. The Nonlinear Optimization with Initial Values Estimation

The nonlinear unconstrained optimization problems described in Equations (18), (30) and (35) can be turned into the problem expressed in Equations (36)–(39), where *f_i_*(***x***) (*i* = 1, 2, 3) is the cost function of the optimization problem corresponding to Equations (18), (30) and (35), ***x****_i_* (*i* = 1, 2, 3) is the parameters to be optimized and *s_i_* (*i* = 1, 2, 3) is the demission of ***x****_i_*.

(36) $$\underset{x_{i}\in R^{s_{i}}}{\min}\frac{1}{2}{\left\|f_{i}\left(x\right)\right\|}^{2}$$

(37) $$x_{1}={\left[K_{a1x}\;K_{a1y}\;K_{a1z}\;K_{a2x}\;K_{a2y}\;K_{a2z}\;K_{a3x}\;K_{a3y}\;K_{a3z}\;E_{ayx}\;E_{azx}\;E_{azy}\;\nabla_{x}\;\nabla_{y}\;\nabla_{z}\right]}^{T}$$

(38) $$x_{2}={\left[K_{g1x}\;K_{g1y}\;K_{g1z}\;K_{g2x}\;K_{g2y}\;K_{g2z}\;E_{gyx}\;E_{gzx}\;E_{gzy}\right]}^{T}$$

(39) $$x_{3}={\left[\varepsilon_{x}^{g}\;\varepsilon_{y}^{g}\;\varepsilon_{z}^{g}\right]}^{T}$$

The Levenberg-Marquardt (LM) algorithm, one of the most popular algorithms for the solution of nonlinear least squares problems, is used in this paper. To implement LM algorithm, Ceres Solver, an open source C++ library to handle large complex optimization problems, is used. For cost function *f*(***x***) that is strongly convex and twice differentiable, the iterative sequence using LM algorithm will be

(40) $$x^{\left(k+1\right)}=x^{\left(k\right)}-{\left(H^{\left(k\right)}+\beta^{\left(k\right)}\mathrm{diag}\left(H^{\left(k\right)}\right)\right)}^{-1}J^{\left(k\right)}$$

where ***x***^(*i*)^ (*i* = *k*, *k*+1) is the parameters vector at the *i*th iteration, ***H*** is the Hessian matrix of *f*(***x***), ***J*** is the Jacobian matrix of *f*(***x***) and *β* is the blending factor that determines the mix between steepest descent and Newton-Raphson [25]. However, the initial values have great influence on the convergence and accuracy of LM algorithm. To ensure the initial values are closer to the optimal solution, the linear scale factors are approximately estimated as the initial values of the optimization, considering that the linear scale factors are more important to navigation-grade IMU in factory calibration. Initial values estimation for linear scale factors before the optimization process is proposed in this paper. Ignoring the nonlinear scale factors, misalignment errors and bias of IMU, the linear scale factors of accelerometer and gyroscope are estimated by the cost functions shown in Equations (41) and (42), respectively.

(41) $$\underset{K_{a1}^{0}}{\min}\frac{1}{2}\sum_{i=1}^{m}{\left\|{\left\|K_{a1}^{0}N_{i}^{a}\right\|}^{2}-{\left\|G^{n}\right\|}^{2}\right\|}^{2}$$

(42) $$\underset{K_{g1}^{0}}{\min}\frac{1}{2}\sum_{i=1}^{n}{\left\|{\left\|K_{g1}^{0}N_{sum}^{i}\right\|}^{2}-{\left\|2\theta \right\|}^{2}\right\|}^{2}$$

where $K_{a1}^{0}$ and $K_{g1}^{0}$ are the rough linear scale factor matrix of accelerometer and gyroscope respectively. Hence, the initial values of linear scale factors can be approximately determined. 

## 5. Simulations and Experiments

### 5.1. Analysis of Simulation Results

Monte Carlo simulations are conducted to verify the proposed method. Using MATLAB, the IMU outputs with random errors are generated in a uniform distribution as the true values. The calibration is conducted using the proposed method, and the calibration errors can be calculated. 

The simulation conditions are set as: the turntable angle errors are 3″, 3′ and 3° (1σ), respectively to verify the relationship between the accuracy of proposed method and the accuracy of turntable. The random bias of accelerometer and gyroscope are 100 *μ*g (1σ) and 0.01 °/h (1σ), respectively. The biases, linear scale factors, second-order scale factors, third-order scale factors and the misalignment errors comply with the uniform distribution U(−10^4^ *μ*g, 10^4^ *μ*g), U(1 *μ*g/pulse, 5 *μ*g/pulse), U(−5 *f*g/pulse^2^, 5 *f*g/pulse^2^), U(−5 *z*g/pulse^3^, 5 *z*g/pulse^3^), U(−5 × 10^−4^, 5 × 10^−4^). The statistical results of 500 Monte Carlo simulations of accelerometers are shown in Table 2. It should be pointed out that the distribution of all parameters shown in Table 2 are set based on the IMU parameters in real experiments.

Table 2 shows that: firstly, the mean values and the root mean squares of calibration errors in Monte Calo experiments are far less than the calibration parameters which means that all calibration parameters of accelerometers can be calibrated accurately. The maximum calibration errors of bias, linear scale factor, second-order scale factor, third-order scale factor and misalignment error are 0.045 *μ*g, −7.49 × 10^−7^ *μ*g/pulse, 2.55 × 10^−3^ *f*g/pulse^2^, 1.96 × 10^−2^ *z*g/pulse^3^ and −6.00 × 10^−4^, respectively.

Secondly, whatever the turntable errors are, the calibration results of the proposed method are almost the same, and the calibration errors of parameters are of the same order of magnitude. Hence, the turntable angle errors have no effects on the calibration errors of proposed method, which means that the proposed method can calibrate all parameters of accelerometers, including the nonlinear scale factors, without relying on the error of the turntable. 

The statistical results of 500 Monte Carlo simulations of gyroscopes are shown in Table 3. Similar to accelerometers, all calibration parameters of gyroscopes can be calibrated accurately without relying on the angle errors of the turntable. The maximum calibration errors of bias, linear scale factor, second-order scale factor and misalignment error are 9.88 × 10^−10^ °/s, −3.38 × 10^−16^ °/s/pulse, −6.21 × 10^−20^ °/s/pulse^2^ and 7.95 × 10^−10^, respectively. The above simulation results verified the correctness of establishing calibration model of IMU with nonlinear scale factor.

**Figure 4:**
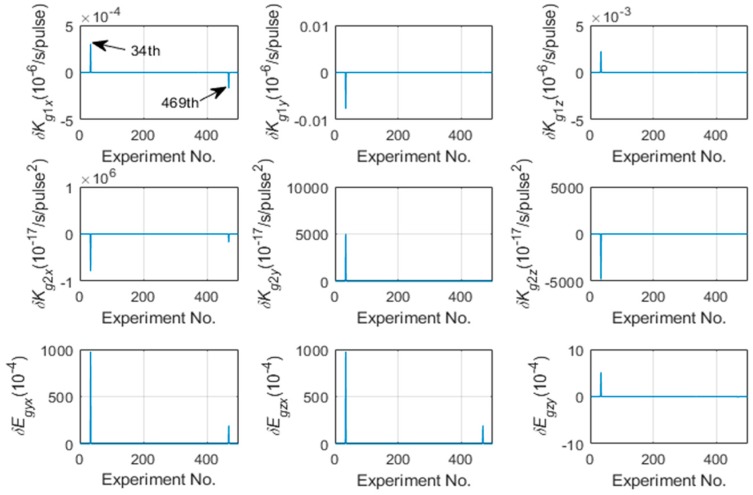
The calibration errors of gyroscope without the initial values estimation.

**Figure 5:**
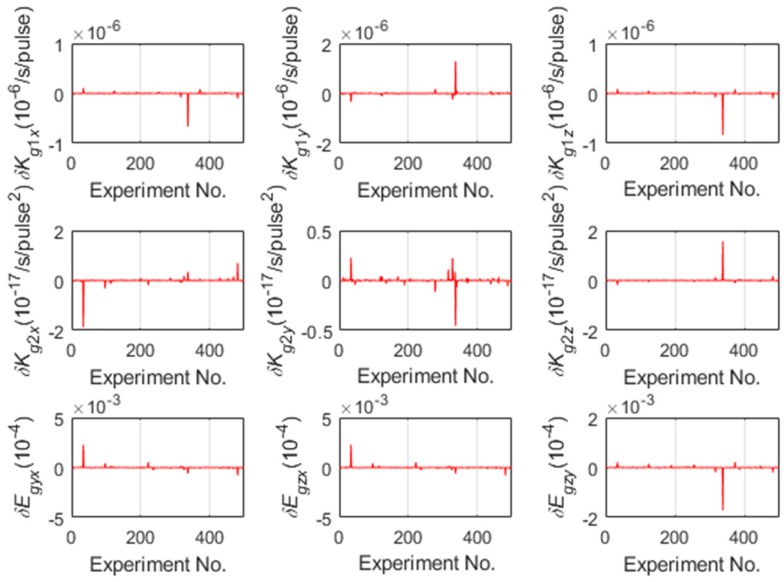
The calibration errors of gyroscope with the initial values estimation.

The calibration results shown in Table 2 and Table 3 are with the initial values estimation proposed in 4.3. To verify that the proposed method can help the LM algorithm converge to the global optimal values, 500 Monte Carlo simulations are carried out, and the calibration errors without and with the initial values estimation of gyroscopes are shown in Figure 4 and Figure 5 respectively.

As shown in Figure 4, the 34th and the 469th Monte Carlo simulation converge to the local optimal values leading to the unacceptable calibration errors because of the incorrect initial values of LM algorithm. Otherwise, the calibration errors with the initial values estimation proposed in 4.3 are acceptable as shown in Figure 5. Figure 4 and Figure 5 shows that estimating the linear scale factors of IMU firstly and make them as the initial values of the optimization can improve the accuracy and the robustness of calibration. The above simulation results showed that the proposed calibration method not only efficiently identified the nonlinear scale factors of IMU without relying on the accuracy of the turntable, but also improved the accuracy and robustness of the calibration with the initial values estimation.

### 5.2. Analysis of Experiment Results

In order to verify the proposed calibration method in practice, the calibration experiments, the la navigation experiments and the moving vehicle experiments are carried out based on 1-axis Rotational Inertial Navigation System (RINS). The RINS consists of three fiber optic gyroscopes, three quartz accelerometers with the precision of 0.01 °/h and 0.01 mg respectively, a rotating mechanism with the rotation axis pointing to vertical direction and a core control processor based on Digital Signal Processor (DSP). 

#### 5.2.1. Calibration Experiments results

**Figure 6:**
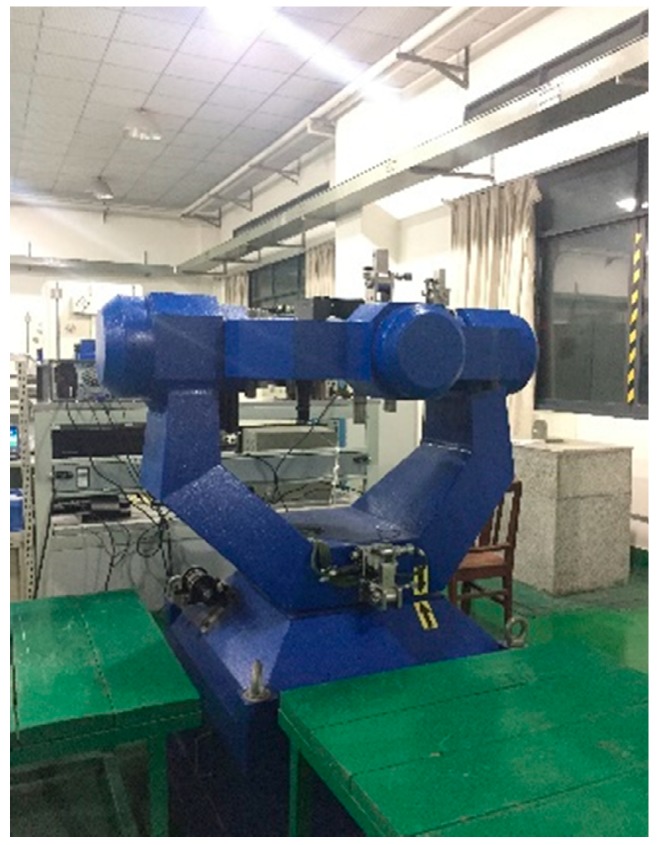
The high-precision 3-axis turntable.

**Figure 7:**
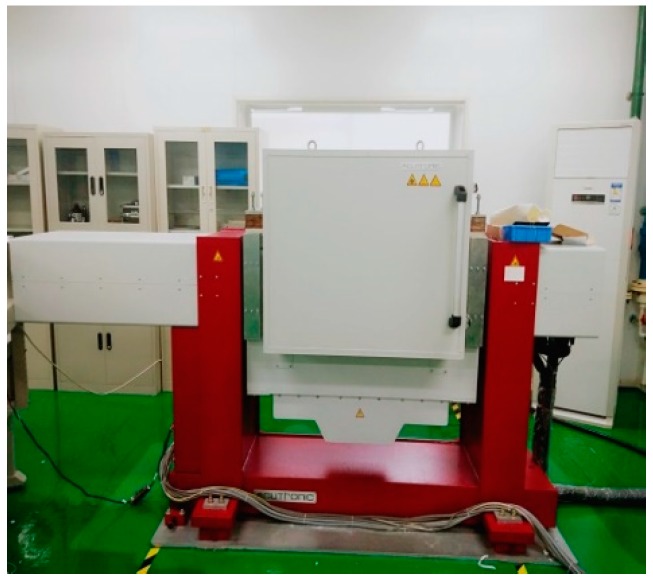
The low-precision 2-axis turntable.

The calibration experiments are conducted using three methods:

(1) The Traditional calibration method based on the high-precision 3-axis turntable shown in Figure 6 with about 5″angle errors, whose accuracy relies on the accuracy of the turntable. 

(2) The Multi-position calibration method in Reference [9] based on the low-precision 2-axis turntable shown in Figure 7 with about 5′angle errors and the rotating mechanism of RINS.

(3) The proposed method in this paper based on the turntable in method 2 and the rotating mechanism of RINS.

It should be pointed out that the rotating mechanism of RINS is used to help the RINS complete the rotation shown in Figure 3 on the 2-axis turntable.

Method 1 calibrates the IMU in the frame of turntable *r*-frame, while method 2 and 3 firstly calibrates the accelerometers and gyroscopes in *p*-frame and *q*-frame respectively, and then calibrates the inter-triad misalignment, making the accelerometers and gyroscopes calibrated to the same orthogonal frame *q*-frame [9]. The inter-triad misalignment $C_{p}^{q}$, the transformation matrix $C_{a}^{r}$ and $C_{g}^{r}$ can be expressed by Euler angles *α*_1_, *β*_1_, *γ*_1_, *α*_2_, *β*_2_, *γ*_2_, *α*_3_, *β*_3_ and *γ*_3_, respectively.

(43) $$C_{p}^{q}=\left[\begin{array}{ccc} cos\alpha_{1}cos\gamma_{1}-sin\alpha_{1}sin\beta_{1}sin\gamma_{1} & cos\gamma_{1}sin\alpha_{1}+cos\alpha_{1}sin\beta_{1}sin\gamma_{1} & -cos\beta_{1}sin\gamma_{1}\\ -cos\beta_{1}sin\alpha_{1} & cos\alpha_{1}cos\beta_{1} & sin\beta_{1}\\ cos\alpha_{1}sin\gamma_{1}+cos\gamma_{1}sin\alpha_{1}sin\beta_{1} & sin\alpha_{1}sin\gamma_{1}-cos\alpha_{1}cos\gamma_{1}sin\beta_{1} & cos\beta_{1}cos\gamma_{1} \end{array}\right]$$

(44) $$C_{a}^{r}=\left[\begin{array}{ccc} cos\alpha_{2}cos\gamma_{2}-sin\alpha_{2}sin\beta_{2}sin\gamma_{2} & cos\gamma_{2}sin\alpha_{2}+cos\alpha_{2}sin\beta_{2}sin\gamma_{2} & -cos\beta_{2}sin\gamma_{2}\\ -cos\beta_{2}sin\alpha_{2} & cos\alpha_{2}cos\beta_{2} & sin\beta_{2}\\ cos\alpha_{2}sin\gamma_{2}+cos\gamma_{2}sin\alpha_{2}sin\beta_{2} & sin\alpha_{2}sin\gamma_{2}-cos\alpha_{2}cos\gamma_{2}sin\beta_{2} & cos\beta_{2}cos\gamma_{2} \end{array}\right]$$

(45) $$C_{g}^{r}=\left[\begin{array}{ccc} cos\alpha_{3}cos\gamma_{3}-sin\alpha_{3}sin\beta_{3}sin\gamma_{3} & cos\gamma_{3}sin\alpha_{3}+cos\alpha_{3}sin\beta_{3}sin\gamma_{3} & -cos\beta_{3}sin\gamma_{3}\\ -cos\beta_{3}sin\alpha_{3} & cos\alpha_{3}cos\beta_{3} & sin\beta_{3}\\ cos\alpha_{3}sin\gamma_{3}+cos\gamma_{3}sin\alpha_{3}sin\beta_{3} & sin\alpha_{3}sin\gamma_{3}-cos\alpha_{3}cos\gamma_{3}sin\beta_{3} & cos\beta_{3}cos\gamma_{3} \end{array}\right]$$

The calibration results of method 1, 2 and 3 for RINS are shown in Table 4. It is obvious that method 3 proposed in the paper can calibrate the nonlinear scale factors of IMU compared with Method 2. Method 1 calibrates the IMU by the comparison of the IMU outputs with the turntable outputs, hence the results of calibration rely on the precision of the turntable. However, the multi-position calibration methods calibrate the IMU in a reference frame instead of the turntable frame. Therefore, the precision of the turntable has no effects on the calibration results.

#### 5.2.2. Navigation Experiments in lab results

Install the RINS on the 3-axis turntable shown in Figure 6, and perform the self-alignment and navigation experiments in 2 states:

(1) The quasi-static state that keeps the turntable in a fixed angle;

(2) The swing state that enables the turntable swing along 3 axes in the condition shown in Table 5, where Heading, Pitch, Roll denotes the rotation axis of the turntable.

**Figure 8:**
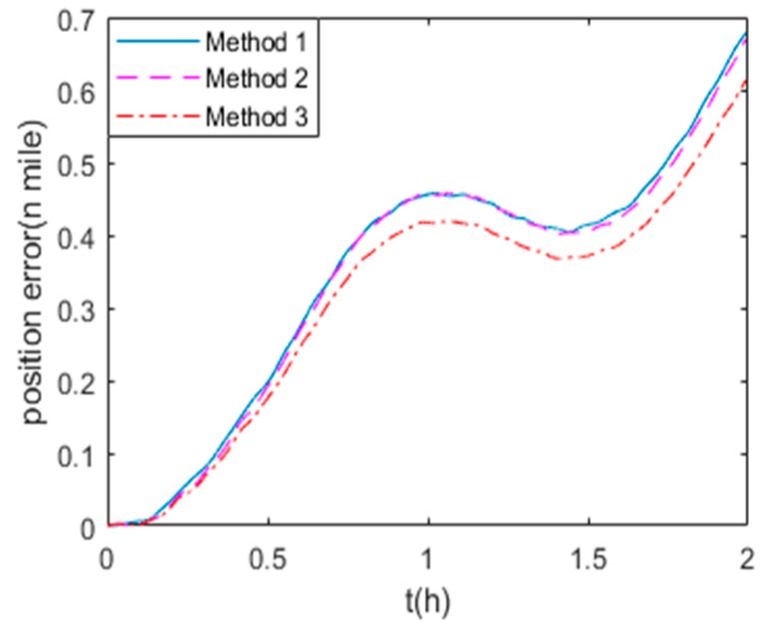
The navigation errors in state (1)**.**

**Figure 9:**
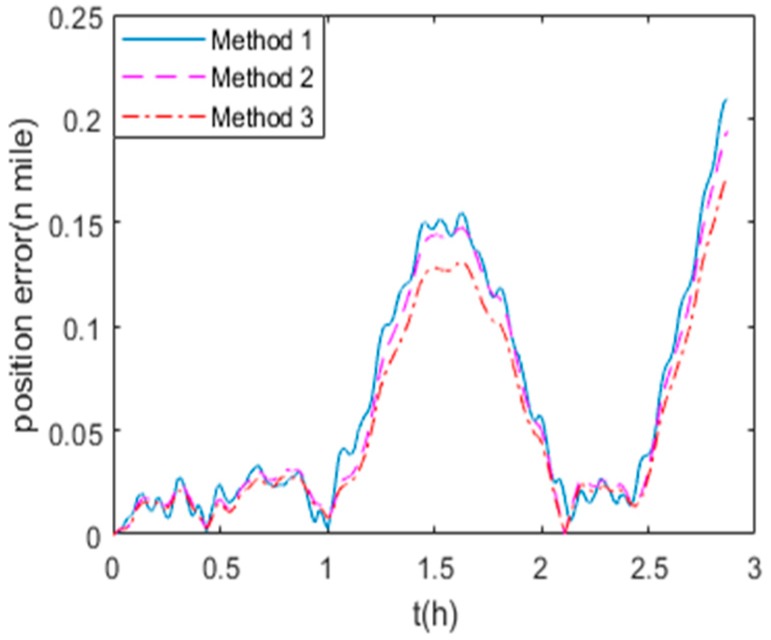
The navigation errors in state (2).

The position errors in state (1) and (2) are shown in Figure 8 and Figure 9 respectively. Method 1, 2 and 3 are the calibration methods described in Section 5.2.1. It is obvious that Method 1 and Method 2 have the similar accuracy on the position errors, while Method 3 proposed in this paper leads to higher precision of navigation for RINS. 

**Figure 10:**
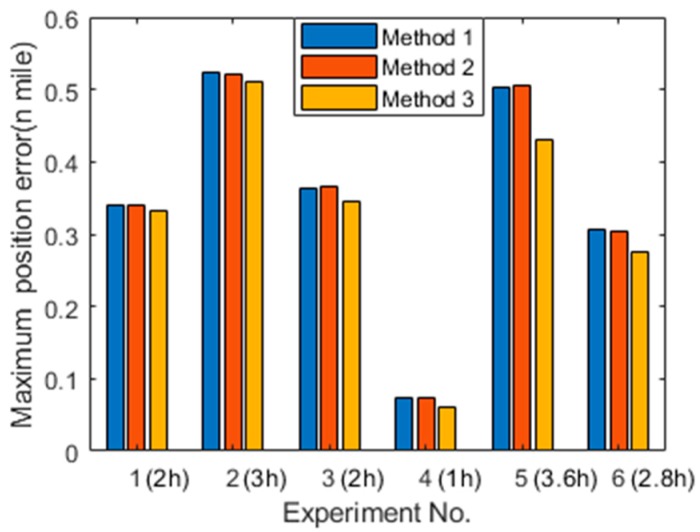
The position errors of multiple navigation experiments.

Multiple navigation experiments in the lab condition are performed to verify the proposed method. The maximum position errors and the navigation time of each experiment are shown in Figure 10. Experiment 1 to 3 are in state (1), while experiment 4 to 6 are in state (2). Compared with Method 1 and Method 2, the position accuracy based on the proposed calibration method 3 can be improved up to 5.19% in quasi-static state and 17.89% in swing state. The position errors can be reduced through the calibration and compensation of the nonlinear scale factor of IMU, because it contributes to the navigation errors as shown in Equations (13)–(16). Besides, it is concluded that compared with the navigation accuracy under quasi-static conditions, the navigation accuracy under the dynamic conditions can be more improved by the proposed method. It is because that the gyroscope only senses the earth rotation rate in quasi-static state, while it also senses the rotation rate shown in Table 5 in swing state. Hence, the non-linearity of gyroscope’s scale factor is more significant in swing state than in quasi-static stare, and it contributes more to the position error in swing state. 

#### 5.2.3. Moving Vehicle Experiment Results

**Figure 11:**
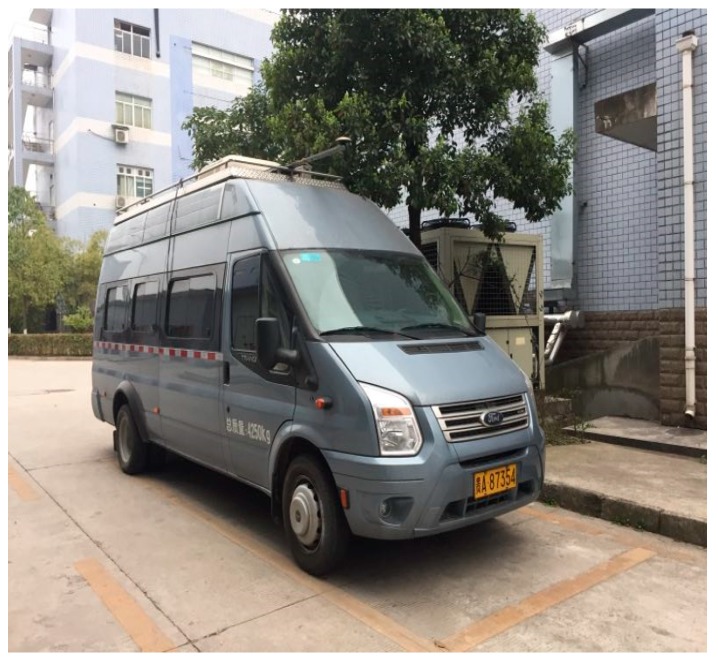
The navigation experiment vehicle.

**Figure 12:**
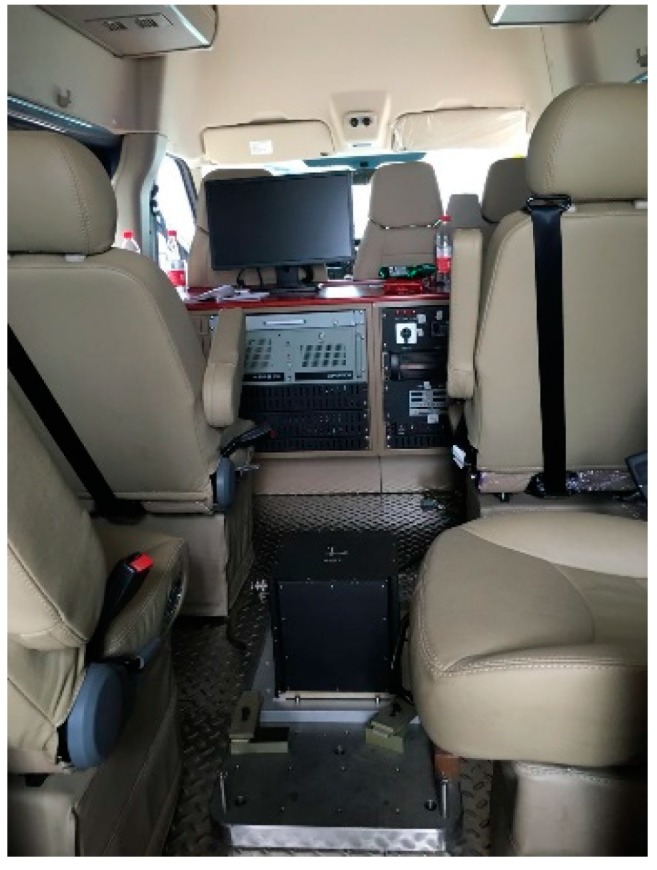
The RINS in navigation experiment vehicle.

**Figure 13:**
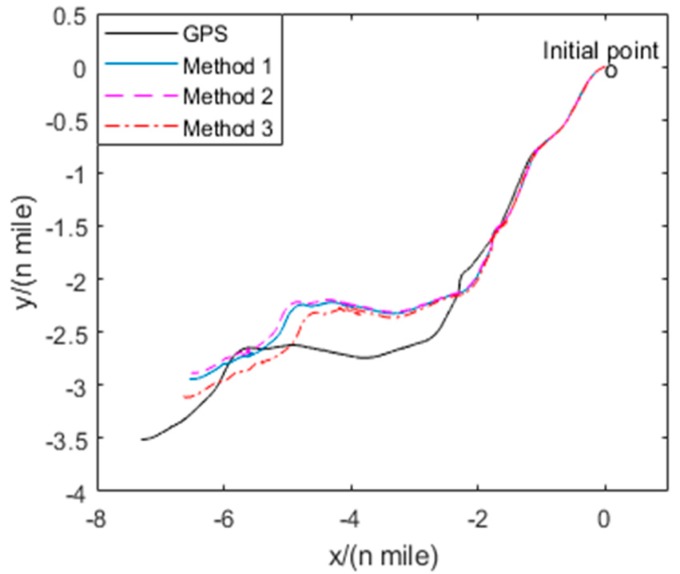
Trajectories of the navigation results.

To verify the calibration method based on the navigation errors of RINS, the moving vehicle experiments are carried out 3 times using the same calibration results to compare the results of three different calibration methods. The navigation experiment vehicle is shown in Figure 11, which is a human operated automobile equipped with a GPS receiver, 1-axis RINS and a computer for data visualization. The 1-axis RINS is installed inside the vehicle as shown in Figure 12. The precision of GPS is 1m, as the reference for navigation. The trajectory of the vehicle is shown in Figure 13, and the route includes movements of turning, uphill, downhill, acceleration and deceleration within 3.2 hours.

**Figure 14:**
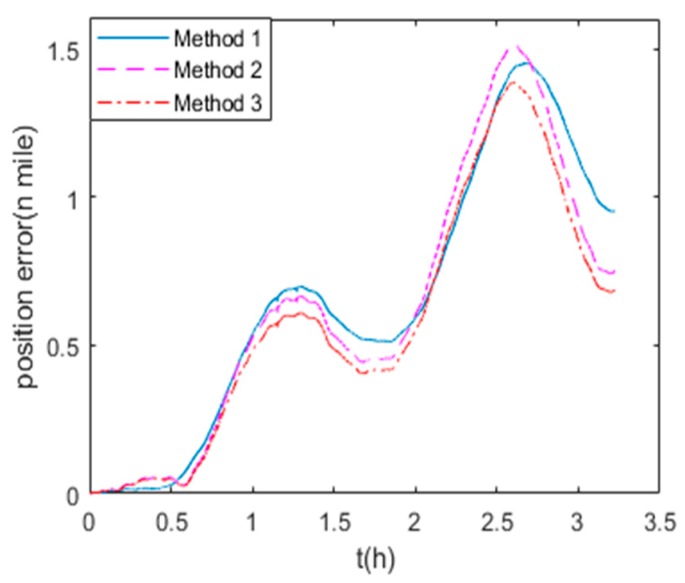
Position errors of three navigation results.

The maximum position errors of the three field experiments are shown in Table 6. Because the results of the three experiments are similar, we take the first experiment result for example for detailed analysis in this paper. The trajectories of the GPS output and the RINS navigation result using the parameters of methods 1–3 are shown in Figure 13. The position errors of three RINS navigation results are compared with the GPS output in Figure 14. The field experiment results show that the precision of method 1 is approximately equal to that of method 2. As the nonlinear scale factors can be accurately calibrated, the navigation results using the parameters of method 3 are better than both method 1 and method 2. As shown in Table 6, the position error of method 3 in moving vehicle navigation experiments can be decreased by 12.19% maximally compared to that of method 1. Besides, the results of three field experiments show that the maximum position error can be reduced by an average of 11% with the calibration and compensation of nonlinear scale factor of IMU. Different from the lab experiments, the accelerometer senses the acceleration of the vehicle in addition to the gravity acceleration in the field experiments. Therefore, the non-linearity of accelerometer’s scale factor is more significant in field experiments than that in lab experiments. Hence, similar to the results of navigation experiments in lab conditions, it can be concluded that the position precision is also improved in field condition using the proposed method, which can estimate the IMU nonlinear scale factors accurately without high-precision turntable. 

## 6. Conclusions

This paper presents a study on the optimized calibration method with nonlinear scale factor for IMU. The effects of nonlinear scale factors of IMU are analyzed, and it proved that the nonlinear scale factors should not be ignored in order to improve the accuracy of navigation for high-precision INS. A nonlinear optimization model of IMU is established, and the optimized calibration motion of IMU is designed based on the analysis of sensitivity of the cost function to the calibration parameters. To solve the nonlinear optimization problems and obtain the global optimal values, LM algorithm of Ceres Solver is used, and in addition, the model for estimating the initial values of nonlinear optimization is established to improve the accuracy and robustness of the optimization. Finally, simulations and experiments are conducted to test the performance of the proposed method. The results of navigation experiments based on the traditional calibration method, the multi-position calibration method without the nonlinear scale factors and the proposed calibration method are compared. It is shown that in the calibration of nonlinear scale factor for IMU without high-precision turntable, the position precisions can be improved by up to 17% in the lab conditions and 12% in the moving vehicle experiment respectively. It is concluded that the traditional calibration method and the multi-position calibration method without the nonlinear scale factors have the similar accuracy, while the proposed method with the nonlinear scale factors leads to higher precision of navigation for INS. 

## Figures and Tables

**Table 1:** The definition of frames.

Symbol	Frames
*i*	The orthogonal inertial frame
*n*	The orthogonal navigation frame directs east-north-up (ENU)
*n′*	The computer navigation frame
*e*	The earth-fixed frame
*r*	The turntable frame
*a*	The non-orthogonal frame denoted by accelerometers’ sensitivity axes
*g*	The non-orthogonal frame denoted by gyroscopes’ sensitivity axes
*p*	The orthogonal frame defined by *a*-frame
*q*	The orthogonal frame defined by *g*-frame

**Table 2:** Statistical results of Monte Carlo simulations for accelerometers.

Calibration Parameters		Distribution	3″ Turntable Angle Errors		3′ Turntable Angle Errors		3° Turntable Angle Errors	
			Mean	RMS	Mean	RMS	Mean	RMS
Bias (*μ*g)	▽_x_	U(−10^4^, 10^4^)	4.32 × 10^−2^	1.90	4.33 × 10^−2^	1.91	4.50 × 10^−2^	2.11
	▽_y_		2.14 × 10^−2^	2.02	2.14 × 10^−2^	2.01	2.31 × 10^−2^	1.95
	▽_z_		−3.69 × 10^−3^	1.96	−3.68 × 10^−3^	1.96	−2.95 × 10^−3^	2.06
Linear scale factor (*μ*g/pulse)	*K_a_*_1*x*_	U(1, 5)	1.17 × 10^−7^	1.90 × 10^−5^	1.17 × 10^−7^	1.90 × 10^−5^	1.16 × 10^−7^	2.04 × 10^−5^
	*K_a_*_1*y*_		−2.37 × 10^−7^	3.35 × 10^−5^	−2.37 × 10^−7^	3.35 × 10^−5^	−2.28 × 10^−7^	3.41 × 10^−5^
	*K_a_*_1*z*_		−7.49 × 10^−7^	1.86 × 10^−5^	−7.48 × 10^−7^	1.86 × 10^−5^	−7.22 × 10^−7^	1.81 × 10^−5^
Second-order scale factor (*f*g/pulse^2^) ^1^	*K_a_*_2*x*_	U(−5, 5)	1.70 × 10^−3^	4.93 × 10^−2^	1.69 × 10^−3^	4.94 × 10^−2^	1.75 × 10^−3^	5.40 × 10^−2^
	*K_a_*_2*y*_		−3.14 × 10^−4^	4.64 × 10^−2^	−3.13 × 10^−4^	4.64 × 10^−2^	−2.86 × 10^−4^	4.52 × 10^−2^
	*K_a_*_2*z*_		2.45 × 10^−3^	4.72 × 10^−2^	2.46 × 10^−3^	4.72 × 10^−2^	2.55 × 10^−3^	4.94 × 10^−2^
Third-order scale factor (*z*g/pulse^3^) ^2^	*K_a_*_3*x*_	U(−5, 5)	1.10 × 10^−3^	0.37	1.10 × 10^−3^	0.38	1.48 × 10^−3^	0.40
	*K_a_*_3*y*_		2.41 × 10^−3^	0.62	2.40 × 10^−3^	0.62	2.12 × 10^−3^	0.63
	*K_a_*_3*z*_		1.96 × 10^−2^	0.37	1.96 × 10^−2^	0.37	1.88 × 10^−2^	0.35
Misalignment errors (10^−4^)	*E_ayx_*	U(−5,5)	−5.25 × 10^−4^	1.91 × 10^−2^	−5.25 × 10^−4^	1.91 × 10^−2^	−5.04 × 10^−4^	1.90 × 10^−2^
	*E_azx_*		−5.87× 10^−4^	1.90 × 10^−2^	−5.87 × 10^−4^	1.90 × 10^−2^	−6.00 × 10^−4^	1.87 × 10^−2^
	*E_azy_*		2.20 × 10^−4^	1.87 × 10^−2^	2.20 × 10^−4^	1.87 × 10^−2^	2.19 × 10^−4^	1.97 × 10^−2^

^1^ 1*f*g (femto g) = 10^−15^g. ^2^ 1*z*g (zepto g) = 10^−21^g.

**Table 3:** Statistical results of Monte Carlo simulations for gyroscopes.

Calibration Parameters		Distribution	3″ Turntable Angle Errors		3′ Turntable Angle Errors		3° Turntable Angle Errors	
			Mean	RMS	Mean	RMS	Mean	RMS
Bias (10^−6^ °/s)	*ε_x_*	U(−5, 5)	6.48 × 10^−4^	1.58 × 10^−2^	6.46 × 10^−4^	1.58 ×10^−2^	5.52 × 10^−4^	1.57 × 10^−2^
	*ε_y_*		9.85 × 10^−4^	1.94 × 10^−3^	9.84 × 10^−4^	1.95 × 10^−3^	9.88 × 10^−4^	1.94 × 10^−3^
	*ε_z_*		8.50 × 10^−4^	2.04 × 10^−3^	8.52 × 10^−4^	2.03 × 10^−3^	8.38 × 10^−4^	2.01 × 10^−3^
Linear scale factor (10^−7^ °/s/pulse)	*K_g_*_1*x*_	U(1, 5)	−7.44 × 10^−10^	3.14 × 10^−8^	1.48 × 10^−9^	1.73 × 10^−8^	4.48 × 10^−10^	1.87 × 10^−8^
	*K_g_*_1*y*_		1.29 × 10^−9^	6.27 × 10^−8^	−3.38 × 10^−9^	4.50 × 10^−8^	−2.48 × 10^−9^	6.51 × 10^−8^
	*K_g_*_1*z*_		−1.24 × 10^−9^	3.88 × 10^−8^	1.47 × 10^−9^	1.90 × 10^−8^	3.51 × 10^−10^	1.68 × 10^−8^
Second-order scale factor (10^−17^ °/s/pulse^2^)	*K_g_*_2*x*_	U(−5, 5)	−1.71 × 10^−3^	9.40 × 10^−2^	−6.21 × 10^−3^	0.15	1.91 × 10^−3^	0.10
	*K_g_*_2*y*_		−9.34 × 10^−5^	2.73 × 10^−2^	1.86 × 10^−3^	2.92 × 10^−2^	2.14 × 10^−3^	6.14 × 10^−2^
	*K_g_*_2*z*_		2.63 × 10^−3^	7.18 × 10^−2^	−2.43 × 10^−3^	3.53 × 10^−2^	7.88 × 10^−5^	1.19 × 10^−2^
Misalignment errors (10^−4^)	*E_gyx_*	U(−5, 5)	2.03 × 10^−6^	1.18 × 10^−4^	7.95 × 10^−6^	1.90 × 10^−4^	−2.92 × 10^−6^	1.41 × 10^−4^
	*E_gzx_*		1.81 × 10^−6^	1.18 × 10^−4^	7.74 × 10^−6^	1.90 × 10^−4^	−3.38 × 10^−6^	1.31 × 10^−4^
	*E_gzy_*		−2.18 × 10^−6^	8.02 × 10^−5^	3.45 × 10^−6^	4.17 × 10^−5^	−4.81 × 10^−7^	1.81 × 10^−5^

**Table 4:** The calibration results of RINS.

Calibration Parameters	Method 1	Method 2	Method 3	Calibration Parameters	Method 1	Method 2	Method 3
▽_x_ (g)	−1.23 × 10^−2^	−1.23 × 10^−2^	−1.21 × 10^−2^	*K_g_*_1*x*_ (°/s/pulse)	5.08 × 10^−7^	5.08 × 10^−7^	5.09 × 10^−7^
▽_y_ (g)	−1.55 × 10^−2^	−1.55 × 10^−2^	−1.54 × 10^−2^	*K_g_*_1*y*_ (°/s/pulse)	5.10 × 10^−7^	5.10 × 10^−7^	5.10 × 10^−7^
▽_z_ (g)	−1.39 × 10^−2^	−1.39 × 10^−2^	−1.37 × 10^−2^	*K_g_*_1*z*_ (°/s/pulse)	5.08 × 10^−7^	5.08 × 10^−7^	5.09 × 10^−7^
*K_a_*_1*x*_ (g/pulse)	2.32 × 10^−6^	2.32 × 10^−6^	2.32 × 10^−6^	*K_g_*_2*x*_ (°/s/pulse^2^)	—	—	−6.35 × 10^−18^
*K_a_*_1*y*_ (g/pulse)	2.25 × 10^−6^	2.25 × 10^−6^	2.26 × 10^−6^	*K_g_*_2*y*_ (°/s/pulse^2^)	—	—	9.00 × 10^−18^
*K_a_*_1*z*_ (g/pulse)	2.18 × 10^−6^	2.18 × 10^−6^	2.18 × 10^−6^	*K_g_*_2*z*_ (°/s/pulse^2^)	—	—	−9.97 × 10^−18^
*K_a_*_2*x*_ (g/pulse^2^)	—	—	−1.53 × 10^−15^	*E_gyx_* (10^−4^)	—	3.58	3.58
*K_a_*_2*y*_ (g/pulse^2^)	—	—	−1.12 × 10^−15^	*E_gzx_* (10^−4^)	—	13.29	13.30
*K_a_*_2*z*_ (g/pulse^2^)	—	—	−1.21 × 10^−15^	*E_gzy_* (10^−4^)	—	−2.09	−2.09
*K_a_*_3*x*_ (g/pulse^3^)	—	—	−5.07 × 10^−21^	*α*_1_ (′)	—	−14.72	−14.72
*K_a_*_3*y*_ (g/pulse^3^)	—	—	−7.80 × 10^−21^	*β*_1_ (′)	—	0.0039	0.0039
*K_a_*_3*z*_ (g/pulse^3^)	—	—	−6.91 × 10^−21^	*γ*_1_ (′)	—	−4.84	−4.84
*E_ayx_* (10^−4^)	—	4.24	4.24	*α*_2_ (′)	−7.48	—	—
*E_azx_* (10^−4^)	—	3.21	3.21	*β*_2_ (′)	−0.69	—	—
*E_azy_* (10^−4^)	—	1.74	1.75	*γ*_2_ (′)	−7.36	—	—
*ε_x_* (°/s)	−3.44 × 10^−6^	−3.44 × 10^−6^	−3.45 × 10^−6^	*α*_3_ (′)	5.97	—	—
*ε_y_* (°/s)	2.04 × 10^−6^	2.04 × 10^−6^	2.04 × 10^−6^	*β*_3_ (′)	−0.026	—	—
*ε_z_* (°/s)	−6.71 × 10^−9^	−6.71 × 10^−9^	−6.70 × 10^−9^	*γ*_3_ (′)	−3.77	—	—

**Table 5:** The swing condition of turntable.

	Heading	Pitch	Roll
Swing frequency (Hz)	2	2	8
Swing amplitude (°)	3	3	5

**Table 6:** The maximum position errors of field experiment.

Experiment Number	Method 1	Method 2	Method 3	The Decreased Percentage ^2^
Experiment 1	1.452 n mile ^1^	1.513 n mile	1.275 n mile	12.19%
Experiment 2	1.537 n mile	1.528 n mile	1.389 n mile	9.63%
Experiment 3	1.426 n mile	1.448 n mile	1.263 n mile	11.43%

^1^ 1 n mile (nautical mile) ≈ 1.852 km. ^2^ The decreased percentage of maximum position error between method 3 and method 1.
